# Bifrontal Epidural Hemorrhage Secondary to Scurvy in a 10-Year-Old Boy

**DOI:** 10.1177/00099228221103975

**Published:** 2022-08-17

**Authors:** Neema Patel, Susan Bessler, James Howard, Ronald Cohen, Bella Doshi

**Affiliations:** 1 UCSF Benioff Children’s Hospital Oakland, Oakland, CA, USA

## Case Report

### Introduction

Scurvy, caused by the prolonged deficiency of vitamin C (ascorbic acid), was once a malady predominantly associated with sailors in the 1600s and 1700s. However, the National Health and Nutrition Examination Survey in 2003-2004 found the overall prevalence of vitamin C deficiency in the United States to be 7.1%, suggesting that this is a more significant contributor to medical illness today than previously recognized.^1^ While in the pediatric population the prevalence is <2% in 6- to 11-year-old children and <4% in adolescents, its frequency is increased in children/adolescents with autism spectrum disorder, many of whom manifest food selectivity and have restricted diets.^1^ The relative rarity often leads to misdiagnosis or delayed diagnosis of scurvy and its complications. In this case, we report a unique presentation of scurvy in a 10-year-old boy with unilateral leg pain and bifrontal epidural hemorrhages.

### Presentation, Diagnosis, and Outcome

A 10-year-old Asian boy with a history of food allergies, anemia, and speech delay presented to the emergency department with 3 weeks of unexplained worsening left knee pain and swelling, intermittent headaches, and diarrhea. There was no obvious trauma history. He was unable to ambulate unassisted, with his mother often carrying him due to debilitating pain. On evaluation, he was hypotensive, with diffuse bilateral lower extremity ecchymoses and limited left knee flexion and extension.

His neurological examination was initially normal. However, while undergoing evaluation, he suddenly developed a severe frontal headache followed by a generalized, tonic-clonic seizure ≤1 minute in duration. He developed asymmetric pupils and became obtunded. He was emergently intubated and treated for seizures and elevated intracranial pressure.

**Figure 1.**
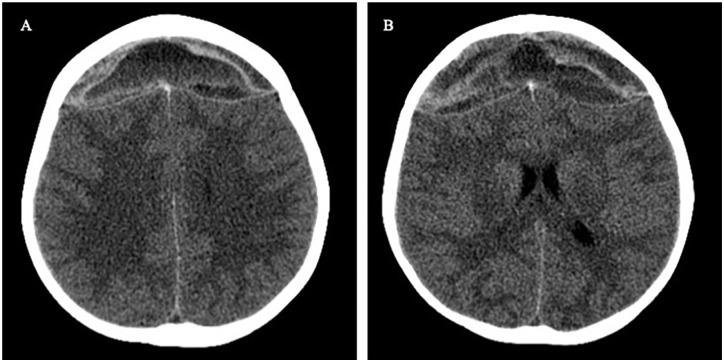
(A and B) Initial axial head computed tomography without contrast demonstrates heterogeneous attenuation in a large bifrontal epidural hematoma, which is consistent with a hyperacute or acute-on-chronic epidural hematoma.

Head computed tomography without contrast revealed a large hyperacute or acute-on-chronic epidural hemorrhage in the bifrontal region with significant mass effect and decreased cerebrospinal fluid space in the ambient cistern (Figure 1). Laboratory studies revealed pancytopenia with hemoglobin of 5.5 g/dL (range, 11.9-14.8 g/dL), hematocrit of 17% (range, 35%-43%), leukocytes of 2.5K/mm^3^ (range, 5.0-12.0K/mm^3^: 44% neutrophils, 47% lymphocytes, 5% monocytes), and platelets of 115K/mm^3^ (range, 150-400K/mm^3^). Peripheral smear was normal. Coagulation profile showed elevated prothrombin time of 19.9 s (normal, 11.7-15.1s), partial thromboplastin time of >200.0 s (normal, 27.0-37.2 s), and international normalized ratio of 1.6 (normal, 0.8-1.2). Comprehensive metabolic panel, lactate dehydrogenase, uric acid, C-reactive protein, and erythrocyte sedimentation rate were within normal limits.

The patient emergently received a bifrontal epidural hematoma evacuation. His coagulopathy resolved after administration of fresh frozen plasma and cryoprecipitate intraoperatively. Initially, he demonstrated no spontaneous movements, with small, fixed, nonreactive pupils, and responded only to noxious stimuli.

Hematology was consulted to evaluate the etiology of his coagulopathy and spontaneous epidural hemorrhages. Factor V, VII, VIII, and IX levels were within normal limits. Factor XI, XII, and XIII levels were low at 58% (range, 66%-137%), 41% (range, 58%-166%), and 59% (69%-143%), respectively. Mild factor deficiencies alone did not appear sufficient to explain his spontaneous intracranial hemorrhages.

Nutritional history revealed a diet consisting exclusively of garlic bread, plain wheat-based noodles, and soy milk for the last 3 years. His height, weight, and body mass index were in the 44th, 55th, and 64th percentiles, respectively, and his growth history was unremarkable. Micronutrient levels were examined. Folate, vitamin B12, and protein-induced vitamin K absence were within normal limits. Several micronutrient levels were low, including vitamin C level of 5 µmol/L (range, 23-115 µmol/L), vitamin A level of 8 µg/dL (range, 26-49 µg/dL), and vitamin D 25-OH level of 10.0 ng/mL (range, 30.0-96.0 ng/mL).

**Figure 2.**
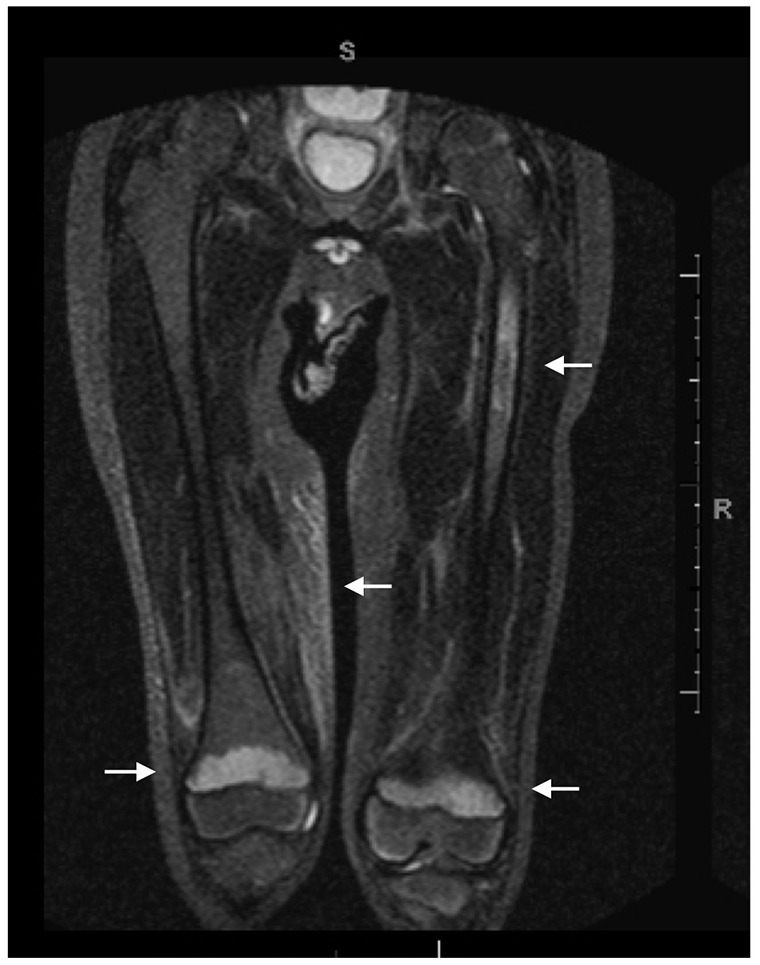
MRI STIR image of bilateral proximal lower extremities which demonstrates an abnormal signal in bilateral metaphyses of distal femurs and shaft of the left proximal femur, and mild subcutaneous edema of the right medial thigh. MRI = magnetic resonance imaging; STIR = short tau inversion recovery.

Given its association with bleeding, joint swelling, and limb pain, vitamin C deficiency became the leading potential explanation for his presentation; additional supportive studies were undertaken. Magnetic resonance imaging (MRI) of the left knee demonstrated marked increased signal in the distal femoral and proximal tibial and fibular metaphyses with displacement of fatty marrow. A 9-cm segment of abnormal signal in the sub-trochanteric bone was noted, with mild subcutaneous edema of the right medial thigh (Figure 2). Dermatological evaluation highlighted cutaneous findings consistent with vitamin C deficiency: ecchymoses in various stages of healing, xerosis and hyperkeratosis, and corkscrew hairs.^2^ Ophthalmologic examination was within normal limits without intraocular hemorrhages. Based on these clinical findings, laboratory results, and imaging studies, scurvy was diagnosed.

The patient received nutritional repletion with vitamin A 20 000 U for 2 days, vitamin C 150 mg twice daily for 1 week, and vitamin D 50 000 U weekly. Repeat levels after 6 weeks were within normal ranges. After correction of his nutritional maladies and resolution of his intracranial bleeding, he was eventually transferred to the inpatient rehabilitation service. His neurological status improved with gradual return of speech and ambulation using a supportive device approximately 12 weeks following treatment.

### Discussion

We report an unusual presentation of a spontaneous bifrontal epidural hemorrhage secondary to vitamin C deficiency or scurvy. Initially, we considered an underlying hematologic disorder precipitating the intracranial hemorrhages given his coagulopathy. However, the coagulopathy easily resolved upon administration of blood products and the mild factor XI, XII, and XIII deficiencies seemed unlikely to account for the severity of his spontaneous intracranial hemorrhages. Oncologic diseases were considered because of the patient’s pancytopenia and leg pain, but the normal peripheral smear and imaging did not support this explanation. Given the patient’s severely limited diet, vitamin levels were sent, revealing profoundly low vitamin C, in addition to low A and D levels.

Vitamin C (L-ascorbic acid or ascorbate) is an essential micronutrient, as humans lack the enzyme gulonolactone oxidase to convert glucose to ascorbic acid.^2,3^ Major contributors of vitamin C are citrus fruits, tomatoes, and potatoes with additional sources including red and green peppers, broccoli, and strawberries.^4^ Pediatric populations at risk of developing scurvy include infants fed boiled or evaporated milk, children exclusively fed meat, and those children with intestinal malabsorption syndromes, end-stage renal disease on chronic hemodialysis, or restricted diet due to neuropsychiatric or developmental disorders including autism or cerebral palsy.^5,6^ Our patient did not have a known condition listed above, but he did have a severely restricted diet lacking in ascorbic acid for multiple years.

Generally, patients often first seek medical attention due to pseudoparalysis, limb pain, and limp, despite having earlier characteristic manifestations including petechiae, ecchymoses, or gingival bleeding. Commonly, there is a delay in diagnosis, as these subtle physical findings occur in a wide spectrum of conditions.^3,7^Numerous studies have demonstrated vitamin C deficiency resulting in these particular musculoskeletal deficits with characteristic radiographic findings. Nonspecific radiographic changes may include generalized osteopenia and cortical thinning. More specific late findings are the scurvy line (area of lucency adjacent to preserved zone of calcification at distal metaphysis due to poorly formed trabeculae), Wimberger ring (increased density outline at the epiphysis), and Pelkan spurs (healing fractures at the periphery of zone of metaphyseal calcification).^1,3,8,9^In our patient, these findings were not present. On MRI, there may be nonspecific multifocal signal abnormalities involving the metaphyses and marrow enhancement, which were seen in our patient and further supported the diagnosis of scurvy.^10,11^

To our knowledge, there is only 1 published case from 2007, which reports cerebral hemorrhage in the setting of vitamin C deficiency. A brain MRI of a 3-year-old boy with left eye proptosis, inability to walk for 2 months, microcytic anemia, and radiographic findings of the right femur consistent with scurvy revealed bilateral extradural hematomas compressing the frontal lobes and a subperiosteal hematoma of the left orbit.^12^ A 1964 study preliminarily investigated the potential role of vitamin C deficiency in adult patients with spontaneous intracranial hemorrhages. Although the study did not demonstrate causation, it highlighted that 75% of 12 patients with bleeding intracranial aneurysms who had low plasma ascorbic content (<0.6 mg%) also had urinary excretion levels below normal (400 mg) following intravenous injection of 1 g of ascorbic acid. Although the mechanism of action in these cases was not elucidated, it was proposed that ascorbic acid plays an integral role in collagen formation of capillary walls and may have contributed to vascular fragility and risk of hemorrhage.^13^

In addition to cerebral hemorrhage, other reported rare complications of scurvy include pulmonary hypertension, cardiac hypertrophy, proptosis due to retroorbital hemorrhage, skeletal muscle degeneration, complex regional pain syndrome, adrenal and bone marrow dysfunction, and alopecia.^14-16^

In our patient, pseudoparalysis with MRI findings of metaphyseal signal abnormality and marrow enhancement and dermatologic changes, including ecchymoses, hyperkeratosis, and corkscrew hairs, are well-known findings of scurvy. Our case emphasizes the rare complication of spontaneous bifrontal epidural hemorrhage secondary to scurvy.

### Conclusion

Scurvy has a spectrum of manifestations beyond its more classic features that can lead to its misdiagnosis or delayed diagnosis. Our case report highlights the rare complication of spontaneous bifrontal epidural hemorrhage secondary to scurvy in a pediatric patient with a restricted diet. Overall, this case report seeks to (1) heighten awareness of various common and unique presentations of scurvy to aid in earlier diagnosis and to (2) highlight the consideration of nutritional assessment and evaluation of micronutrient levels in children with the classic findings of petechiae, nonspecific pain or bone pain, or, less commonly, spontaneous, unexplained intracranial hemorrhages, in the setting of restricted diet.

## Author Contributions

NP: Contributed to conception and design; contributed to data interpretation; drafted the manuscript; gave final approval; agrees to be accountable for all aspects of work ensuring integrity and accuracy.

SB: Contributed to conception and design; contributed to data interpretation; critically revised the manuscript; gave final approval; agrees to be accountable for all aspects of work ensuring integrity and accuracy.

JH: Contributed to conception and design; contributed to data interpretation; critically revised the manuscript; gave final approval; agrees to be accountable for all aspects of work ensuring integrity and accuracy.

RC: Contributed to conception and design; contributed to data interpretation; critically revised the manuscript; gave final approval; agrees to be accountable for all aspects of work ensuring integrity and accuracy.

BD: Contributed to conception and design; contributed to data interpretation; critically revised the manuscript; gave final approval; agrees to be accountable for all aspects of work ensuring integrity and accuracy.
